# Introduction to COMPASS: navigating complexity in public health research

**DOI:** 10.1186/1471-2458-11-S5-S1

**Published:** 2011-11-25

**Authors:** Angela J Taft, Mridula Bandyopadhyay

**Affiliations:** 1 Mother and Child Health Research, La Trobe University, 215 Franklin St, Melbourne, Victoria 3000, Australia

## 

An increasing emphasis on the social determinants of health, a more complicated technological and global world and an understanding that human societies can be conceived as complex adaptive systems, have led public health scholars to take an interest in the science of complexity. Complexity science has been described as the study of complex adaptive systems to see ‘the patterns of relationships within them, how they are sustained, how they self organise and how outcomes emerge’ ([1], p. 3). National or local communities, community health services, general practices and public hospitals embedded in political, economic and cultural contexts all act as complex adaptive systems and shape the behaviours of health care professionals, citizens and patients in sometimes unpredictable ways.

Complex adaptive systems are characterised by ‘non-linearities and discontinuities, aggregate macroscopic patterns rather than causal microscopic events, probabilistic rather than deterministic outcomes and predictions, change rather than stasis’ [2]. The Organisation for Economic Cooperation and Development (OECD) held a global science forum in 2008 that explored the application of complexity science for public policy, drawing an important distinction between complicated systems (such as a car) whose interactions obey precise and knowable rules and for which traditional scientific methods are well suited, and complex systems (e.g. traffic) in which non-linear and collective patterns of behaviour are less predictable [2].

The new interest in complexity science in public health led to an acknowledgement that the more traditional public health methods would need stretching. Better techniques would be needed to explore the adaptability of health care systems and the web of sometimes unpredictable social and economic relations impacting on the resilience or vulnerability of people and communities, and the complex patterns of behaviours that can sustain good health.

The COMPASS multi-disciplinary team of public health scholars have expanded their methods and debated the challenges in addressing complexity in the diversity of studies around women’s health presented in this Special Supplement. COMPASS was funded by the Australian National Health and Medical Research Council (NHMRC) public health research capacity building program to build research capacity for addressing complex questions, settings, interventions and population groups for public health benefit.

This special COMPASS supplement of BMC Public Health ‘Navigating Complexity in Public Health Research’ charts the lessons the author investigators in the COMPASS group learnt when conducting research in women’s health, particularly the myriad factors affecting maternal health in culturally diverse, contemporary Australian society. Emerging papers were presented at a special forum in Melbourne on 25^th^ November 2010 where invited discussants responded to the issues raised in each paper and contributed their own insights. The papers presented here reflect the outcomes of these fruitful dialogues.

The Supplement opens with a short reflective piece addressing the most pressing public health problem in Australia today – that of Aboriginal and Torres Strait Islander health [3]. Beginning at the complex intersection of political history and dispossession with gender and maternal mental health, Tanya Koolmatrie’s methodological reflection discusses the need for treading gently along the research pathway when setting out as both an Insider and Outsider on an Indigenous research journey.

Next, as ethics are a foundation for rigorous public health research, Victoria Palmer and colleagues confront conceptual complexity in ethical practice [4]. Their paper debates the inherent methodological paradoxes and ethical tensions in the use of ‘screening’ as a recruitment method for psychosocial intervention studies among vulnerable populations. They discuss how the application of relational ethics to supplement more traditional ethical paradigms sheds light on the dynamics of the inherent social relations between researchers and participants, and contributes to more comprehensive ethical assessment of complex public health studies.

Rhonda Small and colleagues problematise the concept of ‘social support’ and elicit the lessons from two pragmatic community trials which aimed to implement and evaluate differing approaches to social support and augmenting community networks for mothers [5]. These social ecological interventions were conducted where the overlapping and competing needs of the health care and community systems shaping the work patterns of health care professionals met with the needs of individual mothers. The paper discusses the challenges of achieving health enhancing social connections and why these two interventions had limited success.

The paper by Karalyn McDonald and colleagues focuses debate on the conceptual complexity of ‘risk’ surrounding decision-making when women are pregnant or breastfeeding - ‘the maternal body’ [6]. The authors tease out the tension inherent in public health research between the risks and benefits of medicines from varying stakeholder standpoints and perceptions of the competing needs of the mother, the developing fetus and the baby.

Mridula Bandyopadhyay’s paper illustrates the valuable contribution ethnography makes when more structured methods cannot explain unanticipated consequences of health care system reform to reduce maternal mortality in low income countries [7]. She shows how methodological rigour can be retained; and how methods to uncover emergent social and cultural explanations for ‘non-compliant’ behaviours are potentially transferable from the complex low income country context of maternal health care in West Bengal India, to the care of pregnant South Asian women with gestational diabetes in Australia, if sensitivity to context and flexibility in method can be exercised.

The complexity of research involving multiple relationships, far-reaching collaborations, divergent expectations and various outcomes is highlighted in the paper by Fiona Bruinsma and colleagues [8]. In a retrospective cohort study undertaken to understand the health and psychosocial effects of treating tall adolescent girls with high doses of synthetic oestrogens, the authors describe the methodological complexity involving the maintenance of multiple relationships and collaborations with clinicians and the women treated as adolescent girls. The paper offers guidance to maintaining rigour in the complex politics of long term follow-up studies in public health.

The final paper by Della Forster and colleagues is situated in the complex setting of maternity care [9]. The authors reflect on why a model of midwife-led care provision shown to have positive outcomes in a randomised controlled trial was not retained following the trial, and seek to elucidate reasons for the lack of the model’s sustainability. They contrast this with a more recent trial of a different midwife-led model in the same setting which is continuing after the trial’s cessation. The authors argue that Normalisation Process Theory can inform research evaluation design to take all aspects of the context, including midwives’ working conditions into account during the evaluation of interventions, and that this may help in understanding what will sustain behaviour and organisational change in the complex and dynamic context of hospital-based maternity care.

COMPASS brought together a group of early to mid career researchers for five years to work on addressing questions of complexity in their research undertakings. This Special Supplement is one outcome of our collective endeavours. We hope readers will find useful insights which will contribute to a stronger understanding of how public health research can utilise ‘new tools for finding unanticipated consequences and unrealised opportunities’ in increasingly complex global public health systems for the health benefit of populations [1].

## Competing interests

The authors declare they have no competing interests.
